# The Impact of Greenspace, Walking, and Cycling on the Health of Urban Residents during the COVID-19 Pandemic: A Study of London

**DOI:** 10.3390/ijerph20146360

**Published:** 2023-07-13

**Authors:** Zulfikar Adamu, Oliver Hardy, Asya Natapov

**Affiliations:** 1 School of The Built Environment and Architecture, London South Bank University, 103 Borough Road, London SE1 0AA, UK; 2 School of Architecture, Building and Civil Engineering, Loughborough University, Sir Frank Gibb Building, RT 1.02, West Park, Leicestershire LE11 3TU, UK

**Keywords:** greenspace, physical activity, COVID-19, health, obesity, walking, cycling

## Abstract

Vulnerability to COVID-19 has been linked to public health issues like obesity and physical fitness, which consecutively can be linked to access to urban greenspace. However, the value of greenspaces remains contentious in the literature and unclear in practice. In view of very high COVID-19 mortality rates, we use data from London boroughs to explore the impact of green infrastructure in terms of the size, accessibility, and support of physical activity and healthy lifestyles (e.g., walking and cycling). Results show no significant relationship between the availability of greenspace and the probability of being obese or dying from COVID-19. Cycling once, thrice, or five times weekly was found to improve healthy weight, as does cycling once a month. However, the probability of dying from COVID-19 during lockdowns is correlated to the frequency of walking or cycling as a result of decreased social distancing, while the frequency of walking and cycling is determined by availability and access to greenspace.

## 1. Introduction

With the earth’s population estimated to approach 9 billion by 2050, up to 75% is expected to be living in cities [1]. Such rapid urbanisation combined with contemporary trends in health and wellbeing, e.g., the rise in preventable diseases like diabetes and hypertension, highlights the need for accessible greenspaces for their associated health benefits in urban areas [2,3]. The interest in widely accessible public greenspace and green infrastructure fits into the pattern of a growing demand for improved quality of life (QOL) in general, which is stimulated by increasing incomes and the change in lifestyle [4]. Urban planners, designers, and other built environment stakeholders are facing escalating pressure to find innovative solutions to integrate greenspaces into otherwise densely populated, often polluted, and unpleasant urban areas.

Yet, urban greenspaces cost money to maintain [5,6] and their development is often threatened by factors such as housing shortage and unavailability of land. When green infrastructure is perceived as rundown, it risks being developed upon and having an inadequate understanding of its benefits or the optimum required greenspace size [5]. In fact, several local authorities in the UK have been compelled to privatise many of such public spaces, and the recession brought about by the COVID-19 pandemic could force more of such privatisation [7]. The Heritage Lottery Fund [8] study found that up to half of local park authorities were either contemplating outsourcing the management of greenspaces and parks or even selling them.

Such privatisation measures taken by local authorities cut down free access to greenspaces, which will bring undesirable consequences to the health and wellbeing of urban populations in view of the importance of such spaces to physical activity (PA). This is because PA helps considerably reduce viral infections in general [9,10,11], and with the COVID-19 pandemic being caused by a viral disease, this becomes crucial [12]. Additionally, PA is directly linked to low risks of chronic conditions like coronary heart disease, diabetes, and hypertension, all of which are, interestingly, the most frequently cited underlying conditions that increase COVID-19 mortalities [2]. Other studies show the following: residents of greener neighbourhoods generally have a better perception of their health and report fewer symptoms [13]; living in neighbourhoods with walkable greenspaces leads to more longevity [14]; the elderly benefit significantly from living in areas with greenspaces [15]. As elaborated later on, these health benefits have a strong demographic link. In other words, the value of greenspaces in terms of healthy urban living should increase if lessons are to be learned in a post-COVID-19 world. The challenge is whether existing systems and methodologies enable stakeholders (from built environment professionals to policy makers) to properly capture the impact of greenspaces in cities.

While they may be easier to cost, the value placed on urban green infrastructure is, however, not straightforward. The UK National Ecosystem Accounts [16] and subsequent UK National Ecosystem Assessments Follow-on [17] both tried to address the issue of conflicting methodologies used in greenspace evaluation. Both processes led to what is claimed to be the first comprehensive national guidance [18] aimed at establishing the economic value of a broader range of landscape resources across differing scales, moving away from specific green investments such as trees or parks [19]. Whilst this approach undoubtedly represented progress, it is primarily hinged on economic value to the detriment of other aspects that also need consideration.

A different valuation approach is taken by the UK charity ‘Fields in Trust’ they study on parks and greenspaces uses ‘welfare weighting methodology, allowing for more informed evidence-based policy decisions’ [20]. It also looks to highlight and quantify the intangible benefits brought about by the welfare of individuals made possible by improved access to green infrastructure. This approach brings to light important statistics, such as the ability of green infrastructure to provide a GBP 111 million saving to the UK’s National Health Service (NHS) every year, and helps link greenspaces to the economics of public healthcare [20].

The variety in methods used to assign a monetary value to greenspaces creates inconsistencies within the field, and it would be understandable if policymakers are distrustful of figures or statistics being presented if there is no evidence of a coherent, homogenised process system behind the valuation. In terms of ‘value’, which is often viewed in the binary prism of it being monetary or otherwise, there are certain health benefits of greenspaces (which could be quantified in terms of cost to public health expenditure) that are worth considering.

According to [5], apart from their health benefits to society being underestimated, the financial constraints that greenspaces have to contend with are aggravated by competing public expenditure needs and the political atmosphere in countries like the UK. Against the backdrop of the COVID-19 pandemic, the lockdown imposed nationwide meant a lack of outdoor activity, and the inaccessibility of parks and greenspaces has been detrimental to physical and mental health, with more people having been forced into crowded areas, increasing their risk of contracting the virus [21]. In New York, cars had to be banned in certain streets to enable the sort of social distancing by pedestrians that would have been achievable if open greenspaces were available [7]. Ref. [22] articulated further evidence of the value of greenspaces in maintaining social distancing and attenuating the transmission of COVID-19 and concluded that greenspaces are necessary for building resilience into urban infrastructure.

Consequently, we argue that the relationship between urban greenspaces needs to be better understood in terms of their size and areas, accessibility to resident populations and how they stimulate PA and healthy lifestyles (e.g., walking), particularly in view of very high COVID-19 mortality rates in places like London. The objective of this study is to investigate the impact that greenspaces, walking, and cycling had on the health of urban residents during the COVID-19 pandemic, using London as an example. The fundamental research questions that formed the basis of the research hypotheses to be formulated and tested were conceived as follows:

(1) Considering the link between obesity and PA, how significant is the relationship between the area of (and access to) greenspace available to the public and the probability of being obese in each London borough?
(2) Given the PA-related types of underlying conditions such as obesity, which increase the likelihood of dying from COVID-19, is there a significant relationship between the area of greenspace available to the population of each London borough and the probability of dying from COVID-19?
(3) Given that greenspaces stimulate PA, what is the relation between the frequency of walking and cycling and the proportion of London residents who are of healthy weight or obese?

## 2. Literature Review

### 2.1. The Value and Use of Urban Greenspaces

Greenspaces are part of the modern urban landscape, but stakeholders have interpreted them in different ways, and a standard definition does not exist. The definition by Vivid Economics, a London Report prepared for Greater London Authority [23], excludes canals, rivers, private gardens, agricultural land, golf courses, sports facilities, and landscaped areas around commercial buildings. Greenspace, according to the Parliamentary Office of Science and Technology [24], includes ‘natural or semi-natural areas partially or completely covered by vegetation that occur in or near urban areas’, and these would include parks, woodlands, and allotments. Regardless of the definition, ref. [25] postulate that landscape is perhaps the most complex economic good to value, as it can be made up of an infinite number of configurations in terms of scale, shape, relief, vegetation cover, colour, and human-made features, all of which interact with each other in distinctive permutations.

This debate is crucial for the benefit of architects, planners, urban designers, landscape architects, and contractors who are primarily tasked with designing and developing greenspaces into towns and cities [26,27]. Ref. [28] question how we define value when it comes to a multi-functional landscape because it is dependent on how various groups/people value its many outputs and how end users choose to utilise the space. This is linked to the incompatibility between the recreational and biodiversity values or goals associated with greenspaces [4]. In terms of recreation, urban designers and public health authorities have been warned by [29] that walking should not just be viewed as an embodied activity that is merely a matter of physical bipedalism but rather has important social and cultural ramifications. In this regard, ref. [30] advises that the importance of urban greenspaces to residents transcends the ‘primordial need for nature’, but walking does offer greater benefits when it is performed on a ‘greenspace’ [31], and a quality greenspace can lower mortality rates in deprived areas [32]. In the context of COVID-19 and its consequent lockdown, there are other essential aspects of urban greenspaces in the life of urban residents that need to be understood as overviewed in the subsequent sections [33].

### 2.2. Health Impacts of Greenspaces and Physical Activity on Urban Residents

One of the immeasurable values of urban greenspaces is improved health outcomes. A study of Tucson, Arizona (USA) by [34] has found an association between the frequency of visiting and using a greenspace and its walkability by local residents and how this could increase residents’ PA. Similar findings were found in Hong Kong by [35] where walking behaviour was enhanced by the availability of eye-level greenspaces and parks in the streets. Ref. [3] conducted a study on 1312 people (25–74-year-old adults) to ascertain the link between greenspaces and occurrences of type 2 diabetes and the body mass index of residents in Dortmund, Germany. They concluded that the absence of greenspaces would nearly double the chances of having type 2 diabetes, but this depended on three greenspace indicators: (a) percentage of greenspace per neighbourhood; (b) a population-weighted recreation location quotient for differentiating the accessibility of greenspaces amongst neighbourhoods; (c) individual level of access to greenspaces.

To further buttress the above, a Dutch study on the self-reported health of over 10,000 residents by [13] found that people who live in greener neighbourhoods tend to perceive their health much better and report fewer symptoms, to the extent that increasing the area greenspaces by just 10% was comparable to the reduction in those symptoms that could reduce the longevity by five years. Given the above, it is questionable whether contemporary built environment professionals and other stakeholders involved in the development of greenspaces are sensitised about either the positive health impacts on residents’ QOL or the indirect costs of their negative health outcomes on public expenditure.

**Figure 1:**
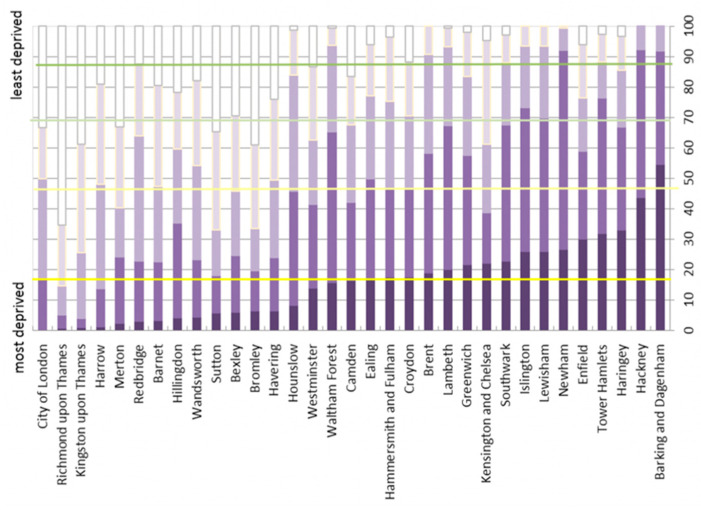
Indices of deprivation for London boroughs as of 2019 [39].

Some of these health impacts have been measured in terms of the rate of survival or life expectancy relative to the availability and accessibility of greenspaces and PA. For instance, in a study of 126 care homes across 17 European cities, access to greenspace was found to be important for the wellbeing of residents [36]; the role of greenspaces in improving the QOL for those suffering from dementia has been emphasised [37]. In a five-year survival study of 3144 senior citizens in Japan, [14] found that those who lived in communities with walkable greenspaces had more longevity, with survival rates higher for those who could easily take strolls near their homes or lived on tree-lined streets and parks. In an urban context and for people living in large cities, the findings of [15] suggest that the elderly, young, and those with secondary education benefitted the most from green areas in their neighbourhood compared to other demographic groups, with the percentage of greenspace in either one- or three-kilometre radius making a significant difference. Ref. [32] found that an increased level of walking was associated with greenspaces, and the link between greenspaces and reduced mortality was observed for deprived areas. It is instructive that the deprived London Boroughs of Newham, Brent, and Hackney (Figure 1) had the highest age-standardised death rates due to COVID-19 [38].

These findings are critical to the debate on the contemporary benefits of greenspaces that stakeholders across various professional callings and interest groups ought to be aware of. This is particularly necessary against the backdrop of the link made between greenspaces and PA as well as those with underlying health issues being susceptible to COVID-19 [9], particularly the risk of deaths [2] due to the ages of elderly persons [39]. Obese individuals have been found to gain more weight or inculcate unhelpful weight management behaviours during the COVID-19 lockdown [40,41] with an additional decline in their mental health [42,43,44] as well as other behavioural addiction disorders [45]. For these vulnerable groups, the medical conclusion drawn by [46] is that preventing an increase in levels of obesity and encouraging PA was as necessary as physical isolation in the fight against COVID-19. Based on recent research focused on trends in Europe, there is an increasing urgency to prioritise greenspaces as a means to address the challenges posed by COVID-19 [47]. In the US, a reduction in COVID-19 mortality was linked to greenspace exposure [11,48,49].

Greenspaces are usually well integrated into the morphology of a city, and their density can be helpful or harmful to residents. On the one hand, lower levels of urban compactness, such as in Stockholm, were found to relate to residents’ being overweight [50]. On the other hand, dense urban neighbourhoods tend to encourage walking, which in turn can reduce levels of obesity and overweight [51]. It is plausible that both extremities (lower compactness or denseness of neighbourhoods) may manifest due to the economic status of city residents, e.g., the rich being able to afford houses with car parking spaces while poorer residents are compelled to walk more often. Nevertheless, there are often municipal factors that shape commuting choices. For example, some cities are now experimenting with non-motorised transportation strategies for enabling green metropolises and eco-cities due in part to concerns about climate change and public health [52]. Although the sharing economy has benefited from such de-motorisation and greening of cities, e.g., via bike sharing programmes such as London Cycle Hire [53], the cycling mode of urban transportation may actually contribute to fomite transmission of COVID-19 [54]. 

In addition, because cyclists using either conventional or electric bikes often share lanes and paths with pedestrians [55], there could be consequences of transmission for both cyclists and pedestrians. Ref. [56] found that the aerodynamics of a fast walking/running or cycling person (assuming they were asymptomatic carriers of the COVID-19 virus) could distribute pathogenic droplets in their wake/trail, making 1.5 m–2.0 m social distancing ineffective, with a distance of at least 5 m being safer. What these findings suggest is that the lifestyle and travel choices of urban residents could inadvertently contribute to their exposure to pandemic viruses like COVID-19. This must be viewed against the backdrop of other factors that contribute to obesity. One of them is socio-economic status [57], which could range from income levels to access to convenience stores [58] to lifestyle choices [59,60].

### 2.3. Importance of Greenspaces to Local Communities

Greenspaces clearly provide many benefits to those in its vicinity. As a consequence, governments and local authorities in cities worldwide have been rolling out greenspaces in otherwise neglected areas of cities, sometimes with mixed responses amongst local residents. In spite of the aforementioned benefits, greenspaces have the propensity to increase rental costs and property values. As a case in point, living within 600 m of a park in London is estimated to increase property value by between 1.9 and 2.9%, with that number increasing to 3–5% for a park deemed to be of ‘high’ quality [61,62]. This can have the unintended consequence of an introduced neighbourhood greenspace causing a ‘green gentrification’ [63] or ‘eco-gentrification’ [64] of an area, leading to the same residents it was intended to benefit becoming ‘priced out’ and eventually displaced. It is nevertheless important for all stakeholders to revisit the urban processes associated with greenspaces and the value placed on them, particularly in the context of COVID-19, its lockdowns and impact on PA, and the overall wellbeing of urban dwellers.

In summary, against the backdrop of the important role of greenspaces in urban neighbourhoods, as well as the COVID-19 pandemic and its impact on people who either have underlying conditions like obesity or who need to be physically active, we examined a number of issues, including the following: (a) whether the area of greenspace available to residents has any impact on the frequency of walking and cycling; (b) if accessibility to open spaces or local parks has an impact on the frequency of walking and cycling; (c) whether the area of available greenspace is linked to the probability of being obese, healthy weight, and/or dying from COVID-19; (d) whether the frequency of walking and cycling could be linked to the probability of dying from COVID-19. Consequently, the objective of this study is to investigate the impact of urban greenspaces and PA on urban residents of London in the context of COVID-19.

## 3. Methodology

**Figure 2:**
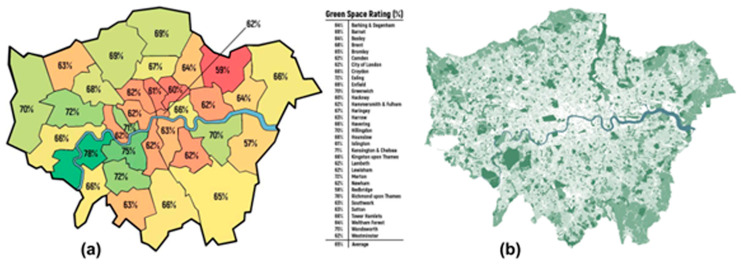
Map showing greenspaces in (**a**) London boroughs and their ranking according to Life Residential, (2009) and (**b**) spatial distribution of greenspaces across Greater London Area (Adapted with permission from Ref. [65]).

This research is aimed at appraising factors, such as availability and access to urban greenspaces in London (Figure 2), as well as resident’s lifestyles, such as walking, cycling, and levels of obesity/overweight, that may have been affected by COVID-19 lockdown and mortality rates. The interwoven nature of these issues required a breakdown of the key issues into distinct hypotheses, as summarised and justified further below.

### 3.1. The Hypotheses and Their Rationale

The likelihood of engaging in PA, such as walking, is connected to the availability of greenspace in urban areas [33,34]. Living in densely populated urban neighbourhoods has also been linked to walking, which can help reduce rates of obesity and overweight [50]. Furthermore, the popularity of bike sharing in London has been found to be significant in promoting physical activity [52]. With these findings in mind, the following two hypotheses were developed: Hypothesis 1 suggests that the area of greenspaces is related to walking and cycling, and Hypothesis 2 proposes that access to such spaces is also related to these activities.

**Hypothesis 1.**  *The area of available greenspace is related to the frequency of walking and cycling*.

**H_0_.**  *The frequency of walking or cycling in a London borough is not determined by the area of greenspace available.*

**H_a_.**  *The frequency of walking or cycling in a London borough is determined by the area of greenspace available.*

**Hypothesis 2.**  *Access to greenspaces, open spaces, or local parks is related to the frequency of walking and cycling*.

**H_0_.**  *The frequency of walking or cycling in a London borough is not determined by the level of access to greenspaces or local parks. (For greenspaces, the ‘Access’ attribute data was derived from [65] open spaces dataset and refers to ‘open, free’ spaces with public access, excluding farmlands and other open spaces outside the London Plan definitions. The travel distance (which differ slightly from London Plan distances) are defined for each open space type as follows: 400 m for open spaces accessible to the public; 400 m for local, small, and pocket parks; 1.2 km for the district; 2.4 km for metropolitan parks; 5 km for regional parks [66]*.

**H_a_.**  *The frequency of walking OR cycling in a London borough is determined by the level of access to greenspaces or local parks.*

It has been shown that the level of individual access to urban greenspaces can help reduce incidences of overweight and type 2 diabetes [3], and increasing the area of greenspace by just 10% leads to significant improvements in those symptoms that could shorten life by 5 years [12]. In addition, lack of PA (e.g., walking) during the COVID-19 lockdown contributed to levels of obesity [39,40], and the reduction in obesity via PA is as necessary as isolation in fighting COVID-19. Consequently, two hypotheses were conceived as follows, focusing on greenspace and probabilities of being obese (Hypothesis 3) and dying from COVID-19 (Hypothesis 4):

**Hypothesis 3.**  *The area of available greenspace is related to the probability of being obese*.

**H_0_.**  *There is no significant relationship between the area of greenspaces available to the public living in London boroughs and the probability of being obese.*

**H_a_.**  *There is a significant relationship between the area of greenspaces available to the public living in London boroughs and the probability of being obese.*

**Hypothesis 4.**  *The area of available greenspace is related to the probability of dying from COVID-19*.

**H_0_.**  *There is no significant relationship between the area of greenspaces available to the public living in London boroughs and the probability of dying from COVID-19.*

**H_a_.**  *There is a significant relationship between the area of greenspaces available to the public living in London boroughs and the probability of dying from COVID-19*.

Finally, considering the aerodynamics of cycling and walking and the spread of aerosolised COVID-19 droplets and how this negates the benefits of 1.5 m social distancing amongst these non-motorised persons [55], Hypothesis 5 was developed. This hypothesis addresses the likelihood of being infected/dying from COVID-19 by virtue of frequent walking and cycling:

**Hypothesis 5.**  *The effect of walking and cycling is related to the probability of dying from COVID-19*.

**H_0_.**  *The probability of a resident dying from COVID-19 in a London borough is not linked to the frequency of walking or cycling*.

**H_a_.**  *The probability of a resident dying from COVID-19 in a London borough is linked to the frequency of walking or cycling.*

### 3.2. Data Collection and Analysis Process

Research datasets for testing our hypotheses were obtained from secondary sources and included greenspace available area and access levels, weekly frequencies of walking and cycling, obesity levels, and COVID-19 deaths in all the London boroughs. These datasets were obtained from government-owned websites. Both sets, i.e., for area/access to greenspaces [66] and for COVID-19 deaths in London boroughs [67,68], were extracted with minimal cleaning and formatting to suit the objectives of this study.

Data for walking, cycling, and obesity levels were collected via the Active People Survey (APS) commissioned by Sport England and is a comprehensive telephone survey of people (16 years and older) who are engaged in sports and active recreation in England. Results from the APS are weighted to ensure representativeness (age by sex, ethnicity, working status by sex, socio-economic classification, and household size) of adults in each local authority.

The data about COVID-19 deaths were collected using 6 June 2020 as a cut-off date, a point when a significant decline in the recorded deaths within London boroughs started to occur. Statistical Package for the Social Sciences (SPSS) was employed for both descriptive and inferential statistics and analysis, which were conducted using a combination of cross-tabulation to provide contingency tables, Chi Square testing, and Fisher’s Exact Statistical Analysis [69,70]. The five hypotheses developed were tested with a validity criterion of each null hypothesis based on a level of significance of 0.05 (i.e., 95% confidence level). Statistical correlation was also used to assess the level of association between walking and cycling frequencies and residents being of healthy weight or obese.

## 4. Results and Discussion

### 4.1. Descriptive Analysis of Data

Greater London is made up of 33 boroughs, which are local administrative areas, each being different in size as well as the area of spaces dedicated as open spaces or local parks that people can use to walk, cycle or do other recreational activities.

#### 4.1.1. Area of Greenspaces

The mean and standard deviation of the area of greenspaces were 18,489.70 and 19,627.31 km^2^, respectively, while the mean and standard deviation of London boroughs were 48,324.83 and 32,770.84, respectively (Table 1). The largest borough (Bromley) was 150,145.1 km^2^, while the smallest (City of London) was 3151.48 km^2^. The boroughs of Bromley, Hillingdon, and Havering have the largest areas of greenspaces (km^2^), while Westminster, the City of London, and Fulham had the smallest area of greenspaces.

#### 4.1.2. Percentage of Greenspaces in London

Havering, Bromley, Richmond upon Thames, Redbridge, and Enfield are examples of boroughs that have the highest percentage of greenspaces in London. The maximum percentage of greenspaces was 58.17%, while the minimum was 5.13%, indicating that there are districts with more than 50% coverage of greenspaces (Table 2). The standard deviation of 12.566 indicates that most of the boroughs had a percentage of greenspaces of approximately between 18% and 42%, which is significant in terms of the area of greenspace in London.

#### 4.1.3. Households’ Access to Open Spaces and Local Parks

**Figure 3:**
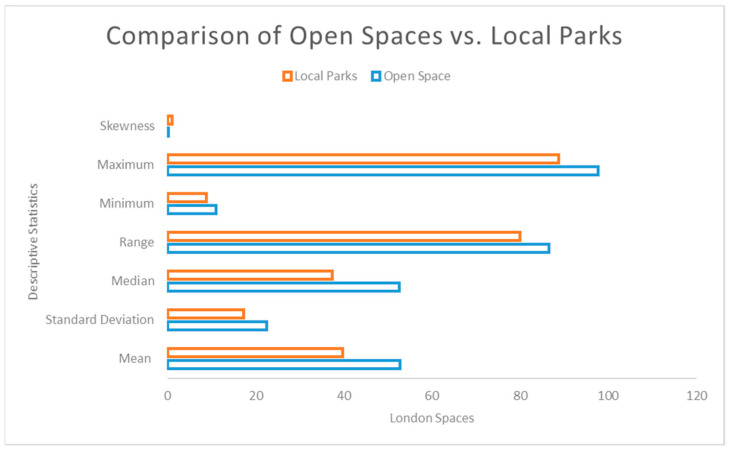
Statistical comparison between Open Spaces and Local Parks.

Households in the City of London Borough have the highest percentage of access to open spaces and local parks at 97.61% and 88.79%, respectively, whereas households with lesser access to these greenspaces are in Redbridge Borough (11.09% access to open spaces) and Wandsworth Borough (8.91% access to local parks). Compared to local parks, (Figure 3) open spaces had higher mean and standard deviation of 52.975 and 22.57, respectively, which means that households in London have better accessibility to open spaces than local parks.

#### 4.1.4. Walking and Cycling Frequency

The walking and cycling data are representative of the proportion of residents that walk for a minimum of 30 min at the following frequencies: once per month, once per week, three times per week, and five times per week. For those who cycle, the data were representative of those who perform any cycling, regardless of the duration or purpose. The largest sample size (1211) was recorded in the Borough of Barnett, while the mean and median sample sizes were 849 and 855 persons, respectively.

**Figure 4:**
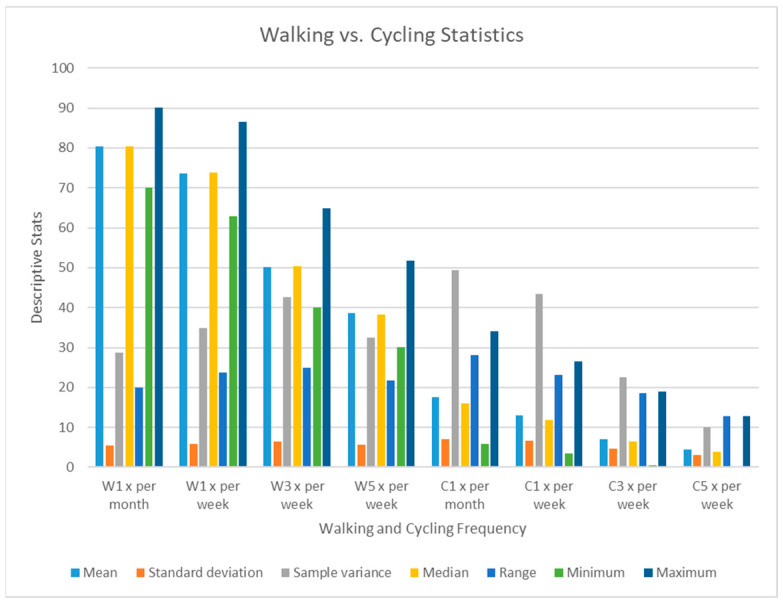
Statistical comparison between frequency of walking and cycling.

From the analysis, London residents tend to use greenspaces more for walking than cycling. As the frequency of walking or cycling increases on a weekly basis, there is a downward trend (Figure 4 from once per month, once per week, and thrice and five times weekly correspondingly. For example, the mean cycling once per month, once per week, three times a week, and five times a week were 17.50, 13.03, 7.01, and 4.55, respectively. In both walking and cycling frequency, the standard deviation was relatively constant, with the highest being 7.025 for cycling once per month, indicating a huge difference in the number of individuals cycling once per month. The minimum and maximum number of individuals walking in open spaces and local parks decreased gradually as the number of walking frequencies increased on a monthly and weekly basis. The significantly fewer number of people walking or cycling five times a week can be attributed to a number of factors such as tight schedules and priority of other activities like workouts/gym sessions in the quest to stay fit.

#### 4.1.5. Obesity

For obesity data, confidence intervals were calculated using a normal approximation for the following classifications: healthy weight, overweight, obesity, and excess weight. For underweight, confidence limits utilised the Wilson Score method due to its suitability for small proportions. The Body Mass Index (BMI) classification covered the following (in kg/m^2^): <18.5 (underweight); 18.5–24.9 (normal weight); 25.0–29.9 (overweight); 30.0–34.9 (class I obesity); 35.0–39.9 (class II obesity); ≥40.0 (class III obesity). The mean sample size for obesity was 597, while the median was 606, and the largest sample size was 862 in the Borough of Barnett.

## 5. COVID-19-Related Deaths

By 6 June 2020, Croydon, Brent, Barnet, and Bromley had the highest number of COVID-19-related deaths at 1514, 1482, 1302, and 1286 mortalities, respectively. The city of London had the least at 18 deaths followed by Richmond and Islington at 413 and 462, respectively (Table 3). The mean number of COVID-19-related deaths was 822 and a standard deviation of 331. The Skewness of 0.267 implies that these deaths were randomly distributed in the boroughs, devoid of other extenuating factors.

### 5.1. Availability and Accessibility of Greenspace and Frequency of Walking and Cycling

The data on walking and cycling frequency were categorized into the following groups, where each person walks for at least 10 min: once per month, once per week, thrice per week, and five times per week. Using the ANOVA technique, the statistical significance of the mean variations was tested in all the walking and cycling frequencies against (a) the area of available greenspaces for Hypothesis 1 and (b) the level of access that residents have to open spaces and local parks for Hypothesis 2.

Using the ANOVA technique to test for the presence of significant variations between the group means required the mean area of greenspace and the mean frequency of walking and cycling in Borough County (Table 4). From the results in the above table, the *p*-value of 0.0000 is less than the significance level of 0.05 used in the analysis test, indicating that we reject Null Hypothesis 1 and accept the alternative statement that the frequency of walking or cycling in London Borough is determined by the area of the greenspace. This means that the more availability of the area of greenspaces, the higher the number of people cycling or walking within a given period of time (week or month). This result agrees with the findings by [32,34] concerning how greenspaces stimulate walking. This funding also provides a positive case for housing the elderly in areas with accessible greenspaces due to the significant benefit to their overall wellbeing [14] and more longevity [13], including for those living in care settings [35] or for those who suffer from dementia [36].

In addition, there is a strong internal correlation between those who walk and those who like to cycle, (*r* = 0.716, *p* > 0.05). Both kinds of activities (and their association) could be borne either due to outdoor lifestyle choices (e.g., recreation) or out of necessity (e.g., commuting to work). Based on the outcome of the hypothesis test using ANOVA (Table 5), the *p*-values (0.00000) are less than the significance level of 0.05; hence, we reject Null Hypothesis 2 and adopt the alternative hypothesis meaning that the frequency at which people walk or cycle in the city of London; the borough is dependent or determined by the level of access to open spaces or local parks. Again, this result agrees with [33,34].

### 5.2. Greenspace Availability and the Probability of Being Obese or Dying from COVID-19

The relationship between the availability of greenspaces in London boroughs and either obesity (Hypothesis 3) or deaths linked to COVID-19 (Hypothesis 4) was explored as outlined in the previous section. The first step in testing each hypothesis required calculating the availability of greenspace itself and the number of deaths, where results indicate that the average area of available greenspaces in the 33 sampled London boroughs is 18,489.76 m^2^ with a standard deviation of 19,627.33 m^2^ (Table 6). The average number of people who died due to COVID-19 (as of 6 June 2020) in the 33 sampled London boroughs was 822 people. Subsequently, results also suggest that the average probability of someone living in the London boroughs being obese is 0.20 (20%) with a standard deviation of 0.05 (5%). The results in Table 3 indicate that there is a weak positive correlation between the availability of greenspaces and the probability of being obese (*r* = 0.297, *p* > 0.05).

**Figure 5:**
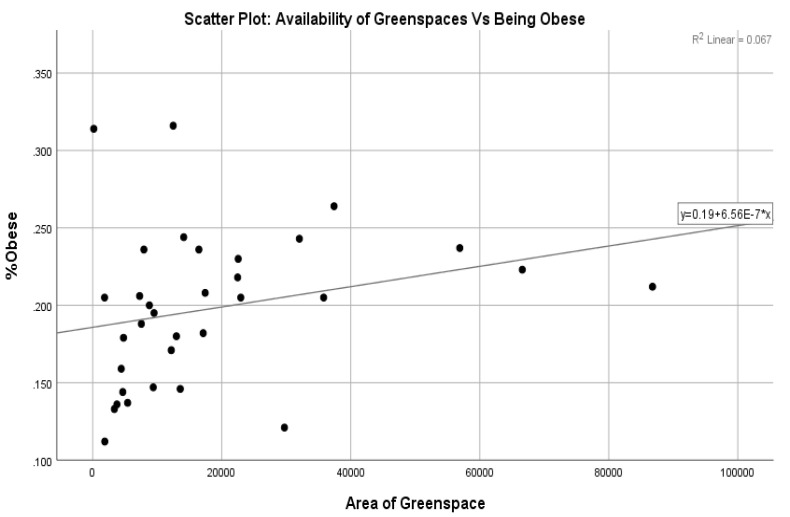
The relationship between the availability of greenspaces and the probability of being obese.

The distribution of the points (Figure 5) in the scatter plot between the availability of greenspaces and the probability of being obese also reveals no relationship between the two variables. Therefore, considering the hypothesis being tested, we have statistical evidence that leads us to accept Null Hypothesis 3 and conclude that there is no significant relationship between the area of greenspaces available to the public living in London boroughs and the probability of being obese.

**Figure 6:**
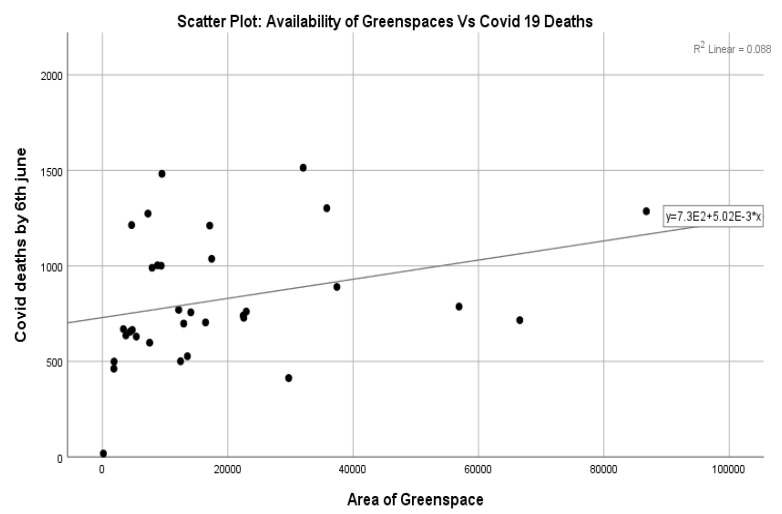
The relationship between the availability of greenspaces and the probability of dying from COVID-19.

Additionally, the correlation results (Table 7) indicated that there was no significant association between the available area of greenspaces and the probability of dying from COVID-19 (*r* = 0.258, *p* > 0.05), i.e., the relationship was a weak positive one. The distribution of the points (Figure 6) also supports this. Therefore, we have statistical evidence to not reject Null Hypothesis 4 and deduce that there is no significant relationship between the area of greenspaces available to the public living in London boroughs and the probability of dying from COVID-19. This result is specifically about the area of greenspaces, and it must be understood in its proper spatial context. In terms of the availability of greenspaces in cities, the COVID-19 lockdown revealed the importance of such spaces [46], showing that accessibility to greenspace leads to a reduction in COVID-19 mortality in the US [48].

**Figure 7:**
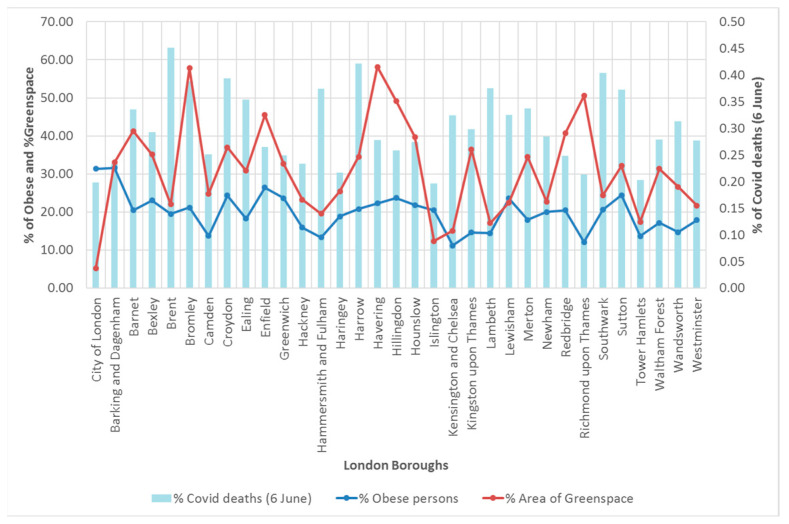
Distribution (in %) of COVID-19 deaths, obese persons, and area of greenspace.

The results from Hypotheses 3 and 4 necessitated not rejecting the two null hypotheses, i.e., there was no significant relationship between the area of greenspaces available and the probability of either being obese or dying from COVID-19. To better understand these results, a closer exploration of the raw data for each borough was conducted. It was evident that there was indeed a stronger pattern in the relationship between the percentage of obese persons and the greenspace areas (Figure 7), but this was not reflected in the percentage of persons who died from COVID-19. In other words, other factors, more significant than the availability of greenspaces, were most likely to play a role in terms of mortality rates of COVID-19 across all London boroughs.

### 5.3. Population Size, Greenspace Availability, and COVID-19 Mortality

**Figure 8:**
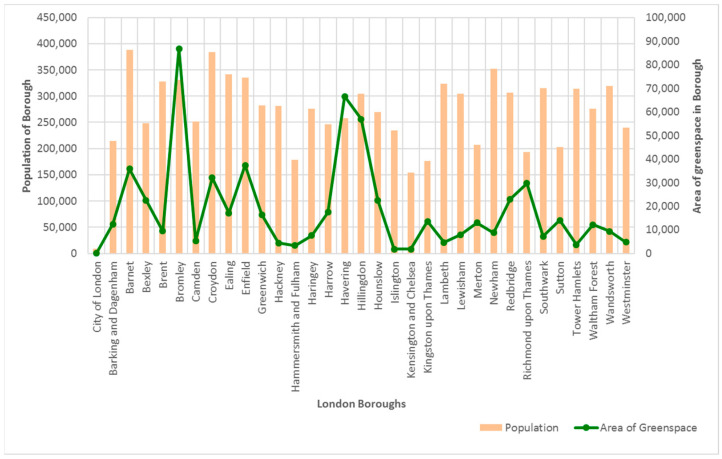
Distribution of greenspace per population of each London borough.

In general, there does not appear to be a distinct proportionate pattern of increase in the availability of greenspace to the population size of each borough (Figure 8). Further examination was conducted to investigate if there was an associated relationship between the population of each borough and the availability of greenspaces using correlation analysis. This analysis was also used to examine the relationship between the availability of greenspaces and the percentage of households that have access to the following four categories of greenspaces: open spaces, local parks, district parks, and metropolitan parks (Table 7).

The results of the correlation exercise (Table 7) revealed that there is a moderate positive correlation between the availability of greenspaces and the population size living in the sampled London boroughs (*r* = 0.323, *p* > 0.05). This indicates that as the population of people living in the borough increases, so does the availability of greenspaces. However, this relationship is not significant.

Additionally, it was found that there is a weak positive correlation between the availability of greenspaces and the percentage of households that have access to district parks (*r* = 0.196, *p* > 0.05). Hence, although the availability of greenspaces increases with the percentage of households that have access to district parks, the relationship is also not significant. On the other hand, a negative correlation was found between the availability of greenspaces and the percentage of households that have access to open spaces (moderately negative: *r* = −0.309, *p* > 0.05), as well as those that have access to metropolitan parks (insignificant negative: *r* = −0.061, *p* > 0.05). This result suggests that (though not significant) as the availability of greenspaces increases, the percentage of households that have access to open spaces and metropolitan parks decreases.

Furthermore, there is a moderate negative relationship between the availability of greenspaces and the percentage of households that have access to local parks (*r* = −0.444, *p* < 0.05). Hence, as the availability of greenspaces increases, the percentage of households that have access to local parks significantly reduces. Since local parks are in close proximity to residential areas, this result suggests that local greenspaces are generally in less populated and probably affluent neighbourhoods, but the link with eco-gentrification processes could not be established.

**Figure 9:**
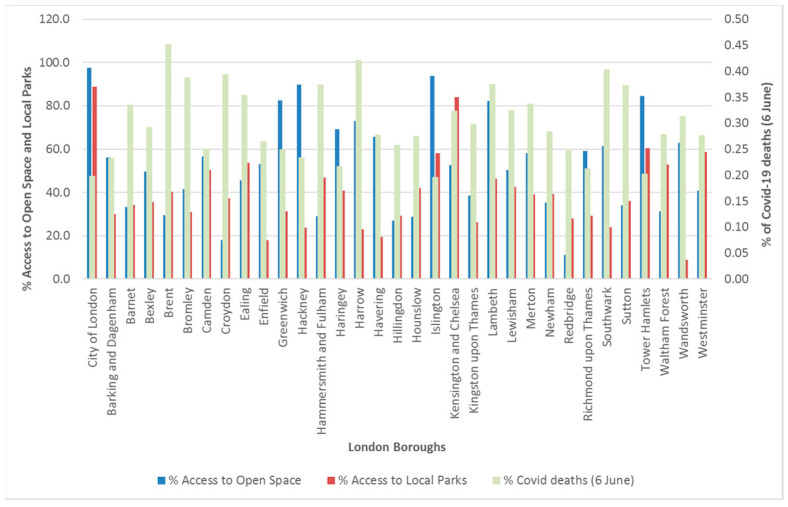
Percentage of households with access to greenspaces and percentage of COVID-19 deaths in each borough.

It may be tempting to contemplate that as more households in a borough had access to greenspaces, e.g., in the form of distant open spaces and local spaces, the mortality rate for that borough would reduce. But this was not the case in general (Figure 9) with the exception of the following boroughs where the percentage of households having access to the greenspaces exceeded the percentage of COVID-19 deaths: City of London, Greenwich, Hackney, Haringey, Islington, and Tower Hamlets. These boroughs are also among the most deprived areas of London (see Figure 1), and their relatively higher death rates have also been found by [37].

### 5.4. Walking and Cycling and Probability of Dying from COVID-19

Hypothesis 5 was aimed at investigating whether the probability of a resident dying from COVID-19 in a London borough is linked to the frequency of walking or cycling or not. The results of the ANOVA test (Table 8) showed that the *p*-value of 0.0000 in the table above is less than the significance level of 0.05 used for the analysis test. Therefore, we reject the null hypothesis and accept that the probability of dying from COVID-19 in a London borough is linked to the frequency of walking or cycling. This discovery presents an intriguing scenario. Previous studies have shown that both walking and cycling are linked to maintaining a healthy lifestyle, which can be beneficial in preventing and combating viral diseases [7,8,9]. These PAs typically require being outdoors, which increases the likelihood of contracting the COVID-19 virus. This finding helps to underscore the importance of the lockdown, social distancing, and isolation as keys to managing the risk of COVID-19. On one hand, it augments previous findings that achieving social distancing in urban areas is easier when walkable areas like greenspaces are available [6]. On the other hand, it also presents a challenge to the medical deduction that PA is as important as isolation in fighting COVID-19, as implied by [45].

It is noteworthy that data on the frequency of walking and cycling do not contain insight into the purpose of such activities, i.e., those who walked/cycled for physical fitness or recreational purposes and those who walked/cycled for work or commuting purposes. Nevertheless, the strong correlation between those who walk and cycle (*r* = 0.716, *p* > 0.05), indicates that those who spent time outdoors for one purpose are likely to do so for the other purpose. This combined likelihood of a pedestrian also being a cyclist could help to explain why the frequency of walking/cycling is linked to higher chances of dying from COVID-19, i.e., the chances of being exposed to the virus are much higher.

### 5.5. The Correlation between Walking, Cycling, Healthy Weight, and Obesity

Based on the analysis results, it was found that there is a positive correlation of 0.5033 between walking once a month and the percentage of individuals with a healthy weight. Conversely, there is a negative correlation of −0.4293 between walking once a month and levels of obesity. This implies that individuals who walk once a month have a higher chance of attaining a healthy weight compared to avoiding obesity. The correlations between walking frequencies (once per month, once per week, thrice per week, and five times a week) and obesity levels were −0.429, −0.411, −0.391, and −0.346, respectively (Table 9). These correlations suggest that there is no significant association between obesity and the frequency of walking. Moreover, there was a nearly identical correlation between achieving a healthy weight and walking at all frequencies. This indicates that regardless of how often a resident walks, as long as they engage in at least 10 min of walking, their likelihood of reaching the desired level of healthy weight increases.

For the cycling frequencies (once per month, once per week, thrice per week, and five times a week), a negative correlation with obesity was found at −0.52, −0.56, −0.567, and −0.572, respectively, indicating that there was no association between cycling and obesity among the participants (Table 10). Obesity could be influenced by other socio-economic factors [57] and lifestyle factors [58,59]. On the other hand, there is a strong positive correlation between cycling once a month and healthy weight at 0.647. Subsequently, the correlations between cycling once per week, thrice per week, and five times a week and healthy weight were 0.674, 0.668, and 0.654, respectively. There was a higher correlation between cycling once per week and thrice per week compared to other frequencies. This is an indication of diminishing returns (for healthy weight) for those who cycle more than thrice a week. Overall, there were strong correlations between healthy weight and cycling, regardless of the frequency of the latter, demonstrating the significant impact of cycling on maintaining a healthy weight.

In summary, the results of the correlation analysis show that there is a relatively higher association between cycling and walking with regard to the percentage of healthy weight. A person who cycles or walks a certain amount of time in a month or week has a higher chance of achieving the ideal healthy weight, but, as shown in the previous section, walking and cycling are not directly associated with obesity.

## 6. Conclusions and Planning Implications

Greenspace has been identified as an important component of healthy urban life and economic prosperity. Greenery is proven to have positive impacts on the health and well-being of individuals and communities living in cities. This study contributes to the debate about the importance and value of greenspaces in the face of the COVID-19 pandemic, which has become a top risk for public health worldwide.

The COVID-19 lockdown period of severe restrictions on citizen activity in the UK and in many other countries was unprecedented. This paper is the first attempt to study the interplay between greenspace and resilience within the urban context and to provide observations on the short-term response to shocks caused by lockdown measures. The COVID-19 pandemic is a ‘once-in-a-lifetime’ global event of utmost importance for planners, local authorities, and built-environment stakeholders in understanding what may happen to our cities in the longer term and in developing appropriate management strategies [71].

Based on the case of London, we showed in this paper that greenspace stimulates walking and cycling. The frequency of walking and cycling in London boroughs is determined by the area of available green infrastructure. People living in places where greenspaces were more available and more accessible tended to walk and cycle more frequently on a weekly basis. We also found that those who walked were more likely to cycle (or vice versa). These underlining relationships between the greenspace, active modes of travel, and recreational activities allow us to quantify a degree of essential physical activity in the healthy lifestyle of urban inhabitants. The promotion of healthy living styles and consecutive changes in people’s behaviour are necessary to improve urban resilience in view of possible future health threats.

At the same time, our results showed that the frequency of walking and cycling can increase the probability of being infected or dying from COVID-19. This link between walking and cycling and COVID-19 mortality needs to be viewed carefully, as, during the studied period lockdown, shielding and self-isolation were among the main means of prevention. Therefore, it is not walking and cycling itself as a physical activity that led to deaths, but extensive outdoor exposure and lack of social distancing associated with them.

It is necessary to prevent possible infections and protect the population, and the considerable impact of such measures is undeniable. Lockdown measures have limited the access of people to services and facilities outside their local areas, whilst lowering the intensity of their usual physical activity. As a result, greenspaces within local neighbourhoods have become more important for outdoor activity than ever. Thinking of improving urban resilience in the face of possible future pandemics, urban planners and authorities need also to consider the wellbeing of citizens, and not just infection rates. Protective measures applied by the authorities have to try maximising physical activity while encouraging other preventive measures, such as distancing and face covering. For these purposes, the layout, size, and functioning of greenspaces gain major importance.

We found that the average probability of being obese for someone living in a London borough is relatively high at 20%. From the perspective of healthy living (walking, cycling, or even jogging), there was no significant association between the availability of greenspaces and the probability of being obese. Although a negative correlation would be desirable (i.e., as more greenspaces became available, people were less obese), the weak positive correlation (*r* = 0.297) suggests a different outcome. There was an additional pattern in the relationship between the percentage of obese persons and the percentage area of greenspace in the borough. The boroughs with more obese people tended to have more area of greenspaces available. These results suggest that, unlike healthy weight, obesity is not directly a function of people’s physical activity. While this specific knowledge may not be new, it is important given the context of COVID-19 mortality rates and the need for vulnerable persons to be involved in physical activity.

Finally, there was also no significant relationship between the area of greenspaces available to the public in London boroughs and the probability of dying from COVID-19. The most deprived boroughs of London had some of the highest COVID-19 deaths, regardless of the amount of greenspace available or accessible to the public. Other factors (including underlying conditions, socio-economic status, and ethnicity), which were not investigated in this study, could be involved.

Future research could investigate deeper these complex relationships between inflectional disease spread and green infrastructure for different types of urban spaces and people activities. Alongside spatial configuration and volumes of usage of the greenspace, it is necessary to also examine other characteristics of usage and human activity, such as repeat or new visitors, to better capture the ways urban green infrastructure contributes to the wellbeing of local communities and to the visiting experience. A comparative understanding of greenspace functioning alongside commuting, for example, becomes particularly timely in the context of the COVID-19 lockdown, whereby a shift from office to working from home is evident.

Our observations suggest the importance of considering greenspace evaluation as a reflection of resilience in a context-specific process, namely, the critical role of the ‘five Ws’ or ‘resilience for whom, what, when, where and why’ [69].COVID-19 mortality rates appear to be influenced by a combination of factors as follows: severe preventive measures adopted by the government nationally; at the city level, pre-existing green conditions and spatial characteristics; at the personal level, citizens’ demographic profile and perceptions of risk. Our results point to the need to develop both national and local strategies, looking again at how to manage health emergency events.

Urban planners, designers, developers, and managers need to consider raising the role of green infrastructure in their future regeneration plans and the impact of associated physical activity patterns. There should be a redirection of energies away from relying on large-scale big parks towards other ways of establishing nature experience, environmental value, and viability maintenance. Given the new focus on social distancing, there is also a clear need for new (more flexible) approaches to how greenery might be valued and integrated both indoors and outdoors.

## Figures and Tables

**Table 1:** Descriptive statistics of Area of Greenspaces compared to Area of Administration.

Area of Greenspace		Area of Admin Geography	
Mean	18,489.7	Mean	48,324.83
Standard Error	3416.676	Standard Error	5704.671
Median	12,476.84	Median	38,676.37
Mode	#N/A	Mode	#N/A
Standard Deviation	19,627.31	Standard Deviation	32,770.84
Sample Variance	3.85 × 10^8^	Sample Variance	1.07 × 10^9^
Kurtosis	4.34768	Kurtosis	2.049358
Skewness	2.03704	Skewness	1.407451
Range	86,613.73	Range	146,993.7
Minimum	161.75	Minimum	3151.48
Maximum	86,775.48	Maximum	150,145.1
Sum	610,160	Sum	1,594,719
Count	33	Count	33

**Table 2:** Descriptive statistics on Percentage of Greenspaces.

Statistics on Percentage of Greenspace	
Mean	30.92156
Standard Error	2.187531
Median	31.36098
Mode	#N/A
Standard Deviation	12.56641
Sample Variance	157.9146
Kurtosis	−0.05243
Skewness	0.363943
Range	53.03921
Minimum	5.132509
Maximum	58.17172
Sum	1020.412
Count	33

**Table 3:** Statistical summary of COVID-19 deaths as of 6 June 2020.

COVID-19 Deaths (as of 6 June 2020)	
Mean	822.3939
Standard Error	57.70412
Median	740
Mode	#N/A
Standard Deviation	331.4849
Sample Variance	109,882.2
Kurtosis	0.165119
Skewness	0.267124
Range	1496
Minimum	18
Maximum	1514
Sum	27,139
Count	33

**Table 4:** Analysis of variation between frequency of walking and cycling and the availability of greenspace.

ANOVA						
Source of Variation	SS ^1^	DF ^2^	MS ^3^	F ^4^	*p*-Value ^5^	F Crit ^6^
Between Groups	9,989,754,451.3153	8	1,248,719,306.4144	29.1733	0.0000	1.970619
Within Groups	12,327,408,819.4354	288	42,803,502.85			
Total	22,317,163,271	296				

^1^ SS is the sum of the square of the residual error in the variations; ^2^ DF is the degrees of freedom (sum of the individual degrees of freedom for each sample); ^3^ MS is the mean squares (the variance due to the differences within individual samples). In this case, the individual samples include the walking and cycling frequencies either weekly or monthly and the area of greenspaces; ^4^ F is the ratio between the sample means and the variations within each group; ^5^ *p*-value is the probability that the observed differences between the variables occurred by chance; ^6^ F crit (critical value) is the measure of variability between the sample groups.

**Table 5:** Analysis of variation between frequency of walking and cycling and access to open spaces and local parks.

Source of Variation	SS	DF	MS	F	*p*-Value	F Crit
Between Groups	216,393.9	9	24,043.77	224.162	0.0000	1.909186
Within Groups	34,323.42	320	107.2607			
Total	250,717.3	329				

SS = sum of squares due to source; DF = degrees of freedom from source; MS = mean sum of squares due to source; F = F statistic.

**Table 6:** Correlation between greenspace availability, obesity, and COVID-19 mortality (*N* = 33).

	*M*	SD	1	2	3
1. Availability of Greenspaces (m^2^)	18,489.76	19,627.33	-		
2. Obese	0.20	0.05	0.297	-	
3. COVID-19 Deaths	822.39	331.49	0.258	−0.014	-

*p* < 0.05.

**Table 7:** Correlation matrix for borough population and access to greenspaces (N = 33).

	Availability of Greenspaces	Population	Open Space	Local Parks	District Parks	Metropolitan Parks
Availability of Greenspaces	-					
Population	0.323	-				
Open Space	−0.309	−0.326	-			
Local Parks	−0.444 *	−0.481 *	0.186	-		
District Parks	0.196	0.478 *	−0.084	−0.450 *	-	
Metropolitan Parks	−0.061	0.036	−0.127	0.091	−0.059	-

* *p* < 0.05.

**Table 8:** Analysis of variation between frequency of walking and cycling and probability of dying from COVID-19.

Source of Variation	SS	DF	MS	F	*p*-Value	F Crit
Rows	2115.029	32	66.09466	5.479582	0.0000	1.804482
Columns	65,101.77	1	65,101.77	5397.266	0.0000	4.149097
Error	385.9837	32	12.06199			
Total	67,602.79	65				

SS = sum of squares due to source; DF = degrees of freedom from source; MS = mean sum of squares due to source; F = F statistic.

**Table 9:** The correlation between walking, healthy weight, and obesity.

	W1 × per Month	W1 × per Week	W3 × per Week	W5 × per Week	% Healthy Weight	% Obese
W1 × per month	1					
W1 × per week	0.97013	1				
W3 × per week	0.875397	0.931843	1			
W5 × per week	0.753205	0.828478	0.937106	1		
% Healthy weight	0.503293	0.513072	0.524669	0.46817	1	
% Obese	−0.42932	−0.4112	−0.39093	−0.34644	−0.86591	1

**Table 10:** The correlation between cycling, healthy weight, and obesity.

	C1 × per Month	C1 × per Week	C3 × per Week	C5 × per Week	% Healthy Weight	% Obese
C1 × per month	1					
C1 × per week	0.977436	1				
C3 × per week	0.916926	0.957273	1			
C5 × per week	0.844844	0.898057	0.960861	1		
% Healthy weight	0.647396	0.674238	0.667966	0.654346	1	
% Obese	−0.52011	−0.56497	−0.56728	−0.57252	−0.86591	1

## Data Availability

Not applicable.

## References

[1] Armour T., Armour S., Hargrave J., Revell T. (2014). Cities Alive: Rethinking Green Infratructure.

[2] Lippi G., Henry B.M., Sanchis-Gomar F. (2020). Physical inactivity and cardiovascular disease at the time of coronavirus disease 2019 (COVID-19). Eur. J. Prev. Cardiol..

[3] Müller G., Harhoff R., Rahe C., Berger K. (2018). Inner-city green space and its association with body mass index and prevalent type 2 diabetes: A cross-sectional study in an urban German city. BMJ Open.

[4] Heide C., Heijman W. (2014). The Economic Value of Landscapes.

[5] Lee A.C., Jordan H.C., Horsley J. (2015). Value of urban green spaces in promoting healthy living and wellbeing: Prospects for planning. Risk Manag. Healthc. Policy.

[6] Askew J., King L., McClymont K., Roberts H., Sinnett D., Smith N. (2011). Green Infrastructure in Urban Areas, Royal Institution of Chartered Surveyors. https://communities.rics.org/gf2.ti/f/200194/18045157.1/PDF/-/RICS_Green_infrastructure_in_urban_areas_1_.pdf.

[7] Samuelsson K., Barthel S., Colding J., Macassa G., Giusti M. (2020). Urban Nature as a Source of Resilience during Social Distancing Amidst the Coronavirus Pandemic. OSF Preprint. https://osf.io/3wx5a/.

[8] Heritage Lottery Fund (2016). State of UK Public Parks. https://www.heritagefund.org.uk/sites/default/files/media/attachments/state_of_uk_public_parks_2016_final_for_web%281%29.pdf.

[9] Jurak G., Morrison S.A., Leskošek B., Kovač M., Hadžić V., Vodičar J., Truden P., Starc G. (2020). Physical activity recommendations during the coronavirus disease-2019 virus outbreak. J. Sport Health Sci..

[10] Romeo J., Wärnberg J., Pozo T., Marcos A. (2010). Physical activity, immunity and infection. Proc. Nutr. Soc..

[11] Martin S.A., Pence B.D., Woods J.A. (2009). Exercise and respiratory tract viral infections. Exerc. Sport Sci. Rev..

[12] Li S., Wang Y., Xue J., Zhao N., Zhu T. (2020). The Impact of COVID-19 Epidemic Declaration on Psychological Consequences: A Study on Active Weibo Users. Int. J. Environ. Res. Public Health.

[13] De Vries S., Verheij R.A., Groenewegen P.P., Spreeuwenberg P. (2003). Natural environments—Healthy environments? An exploratory analysis of the relationship between greenspace and health. Environ. Plan. A.

[14] Takano T., Nakamura K., Watanabe M. (2002). Urban residential environments and senior citizens’ longevity in megacity areas: The importance of walkable green spaces. J. Epidemiol. Community Health.

[15] Maas J., Verheij R.A., Groenewegen P.P., De Vries S., Spreeuwenberg P. (2006). Green space, urbanity, and health: How strong is the relation?. J. Epidemiol. Community Health.

[16] UKNEA (2014). National Ecosystem Assessment. The UK National Ecosystem Assessment: Synthesis of the Key Findings.

[17] UKNEAFO (2014). National Ecosystem Assessment Follow On; The UK National Ecosystem Assessment: Synthesis of the Key Findings.

[18] UNEP-WCMC (2011). UK National Ecosystem Assessment: Understanding Nature’s Value to Society. Synthesis of Key Findings.

[19] Mell I.C., Henneberry J., Hehl-Lange S., Keskin B. (2013). Promoting urban greening: Valuing the development of green infrastructure investments in the urban core of Manchester, UK. Urban For. Urban Green..

[20] Fields in Trust (2018). ‘Revaluing Parks and Green Spaces: Measuring Their Economic and Wellbeing Value to Individuals’. Fields in Trust Website. www.fieldsintrust.org/research.

[21] Freeman S., Eykelbosh A. (2020). COVID-19 and Outdoor Safety: Considerations for Use of Outdoor Recreational Spaces.

[22] Venter Z., Barton D., Figari H., Nowell M. (2020). Urban Nature in a Time of Crisis: Recreational Use of Green Space Increases during the COVID-19 Outbreak in Oslo, Norway (No. kbdum). Center for Open Science. https://osf.io/preprints/socarxiv/kbdum/download.

[23] Vivid Economics (2017). Natural Capital Accounts for Public Green Space in London Report Prepared for Greater London Authority, National Trust and Heritage Lottery Fund Accessed 19/01/19. www.london.gov.uk/sites/default/files/11015viv_natural_capital_account_for_london_v7_full_vis.pdf.

[24] POST (2016). Parliamentary Office of Science and Technology POSTnote 538 October 2016, Green Space and Health. https://www.parliament.uk/postnotes.

[25] Garrod G., Willis K.G. (1999). Economic Valuation of the Environment: Methods and Case Studies.

[26] French S.K., Giacinti J.A., Robinson S.J., Pearl D.L., Jardine C.M. (2022). The urban myth: A lack of agreement between definitions of urban environments used in wildlife health research may contribute to inconsistent epidemiological findings. Urban Ecosyst..

[27] Seitz B., Buchholz S., Kowarik I., Herrmann J., Neuerburg L., Wendler J., Winker L., Egerer M. (2022). Land sharing between cultivated and wild plants: Urban gardens as hotspots for plant diversity in cities. Urban Ecosyst..

[28] Haines-Young R., Potschin M., Brandt J., Vejre H. (2004). Valuing and Assessing of Multifunctional Landscapes: An Approach Based on the Natural Capital Concept.

[29] Green J. (2009). ‘Walk this way’: Public health and the social organization of walking. Soc. Theory Health.

[30] Carpenter M. (2013). From ‘healthful exercise’to ‘nature on prescription’: The politics of urban green spaces and walking for health. Landsc. Urban Plan..

[31] Barton J., Hine R., Pretty J. (2009). The health benefits of walking in greenspaces of high natural and heritage value. J. Integr. Environ. Sci..

[32] Lachowycz K., Jones A.P. (2014). Does walking explain associations between access to greenspace and lower mortality?. Soc. Sci. Med..

[33] Yang Y., Lu Y., Jiang B. (2022). Population-weighted exposure to green spaces tied to lower COVID-19 mortality rates: A nationwide dose-response study in the USA. Sci. Total Environ..

[34] Zuniga-Teran A.A., Stoker P., Gimblett R.H., Orr B.J., Marsh S.E., Guertin D.P., Chalfoun N.V. (2019). Exploring the influence of neighborhood walkability on the frequency of use of greenspace. Landsc. Urban Plan..

[35] Lu Y., Sarkar C., Xiao Y. (2018). The effect of street-level greenery on walking behavior: Evidence from Hong Kong. Soc. Sci. Med..

[36] Artmann M., Chen X., Iojă C., Hof A., Onose D., Poniży L., Lamovšek A.Z., Breuste J. (2017). The role of urban green spaces in care facilities for elderly people across European cities. Urban For. Urban Green..

[37] Mmako N.J., Courtney-Pratt H., Marsh P. (2020). Green spaces, dementia and a meaningful life in the community: A mixed studies review. Health Place.

[38] Harris R. (2020). Exploring the neighbourhood-level correlates of COVID-19 deaths in London using a difference across spatial boundaries method. Health Place.

[39] London Data Store (2020). Indices of Deprivation 2019 Initial Analysis. https://data.london.gov.uk/blog/indices-of-deprivation-2019-initial-analysis/.

[40] Clark A., Jit M., Warren-Gash C., Guthrie B., Wang H.H., Mercer S.W., Sanderson C., McKee M., Troeger C., Checchi F. (2020). Global, regional, and national estimates of the population at increased risk of severe COVID-19 due to underlying health conditions in 2020: A modelling study. Lancet Glob. Health.

[41] Pellegrini M., Ponzo V., Rosato R., Scumaci E., Goitre I., Benso A., Belcastro S., Crespi C., De Michieli F., Ghigo E. (2020). Changes in weight and nutritional habits in adults with obesity during the “lockdown” period caused by the COVID-19 virus emergency. Nutrients.

[42] Robinson E., Gillespie S., Jones A. (2020). Weight-related lifestyle behaviours and the COVID-19 crisis: An online survey study of UK adults during social lockdown. Obes. Sci. Pract..

[43] Mazza C., Ricci E., Biondi S., Colasanti M., Ferracuti S., Napoli C., Roma P. (2020). A Nationwide Survey of Psychological Distress among Italian People during the COVID-19 Pandemic: Immediate Psychological Responses and Associated Factors. Int. J. Environ. Res. Public Health.

[44] Robinson E., Boyland E., Chisholm A., Harrold J., Maloney N.G., Marty L., Mead B.R., Noonan R., Hardman C.A. (2020). Obesity, eating behavior and physical activity during COVID-19 lockdown: A study of UK adults. Appetite.

[45] Zhang Y., Zheng F.M. (2020). Impact of the COVID-19 Pandemic on Mental Health and Quality of Life among Local Residents in Liaoning Province, China: A Cross-Sectional Study. Int. J. Environ. Res. Public Health.

[46] Lippi G., Henry B.M., Bovo C., Sanchis-Gomar F. (2020). Health risks and potential remedies during prolonged lockdowns for coronavirus disease 2019 (COVID-19). Diagnosis.

[47] Katsoulis M., Pasea L., Lai A., Dobson R.J., Denaxas S., Hemingway H., Banerjee A. (2021). Obesity during the COVID-19 pandemic: Cause of high risk or an effect of lockdown? A population-based electronic health record analysis in 1 958 184 individuals. Public Health.

[48] Kleinschroth F., Kowarik I. (2020). COVID-19 crisis demonstrates the urgent need for urban greenspaces. Front. Ecol. Environ..

[49] Marconi P.L., Perelman P.E., Salgado V.G. (2022). Green in times of COVID-19: Urban green space relevance during the COVID-19 pandemic in Buenos Aires City. Urban Ecosyst..

[50] Ståhle A., Caballero L. (2010). Greening metropolitan growth: Integrating nature recreation, compactness and spaciousness in regional development planning. Int. J. Urban Sustain. Dev..

[51] Frank L.D., Andresen M.A., Schmid T.L. (2004). Obesity relationships with community design, physical activity, and time spent in cars. Am. J. Prev. Med..

[52] Russette H., Graham J., Holden Z., Semmens E.O., Williams E., Landguth E.L. (2021). Greenspace exposure and COVID-19 mortality in the United States: January–July 2020. Environ. Res..

[53] Ding H., Sze N.N., Li H., Guo Y. (2021). Effect of London cycle hire scheme on bicycle safety. Travel Behav. Soc..

[54] Dietz L., Horve P.F., Coil D., Fretz M., Eisen J., Van Den Wymelenberg K. (2020). 2019 Novel Coronavirus (COVID-19) Pandemic: Built Environment Considerations to Reduce Transmission. Msystems.

[55] Mayer A. (2020). Motivations and barriers to electric bike use in the US: Views from online forum participants. Int. J. Urban Sustain. Dev..

[56] Blocken B., Malizia F., van Druenen T., Marchal T. (2020). Towards Aerodynamically Equivalent COVID19 1.5 m Social Distancing for Walking and Running. Questions and Answers. Website Bert Blocken, Eindhoven University of Technology (The Netherlands) and KU Leuven (Belgium). http://www.urbanphysics.net/COVID19.html.

[57] Norte A., Sospedra I., Ortíz-Moncada R. (2019). Influence of economic crisis on dietary quality and obesity rates. Int. J. Food Sci. Nutr..

[58] Johnson K.A., Showell N.N., Flessa S., Janssen M., Reid N., Cheskin L.J., Thornton R.L. (2019). Do neighborhoods matter? A systematic review of modifiable risk factors for obesity among low socio-economic status Black and Hispanic children. Child. Obes..

[59] Kopp W. (2019). How western diet and lifestyle drive the pandemic of obesity and civilization diseases. Diabetes Metab. Syndr. Obes. Targets Ther..

[60] Wadden T.A., Webb V.L., Moran C.H., Bailer B.A. (2012). Lifestyle modification for obesity: New developments in diet, physical activity, and behavior therapy. Circulation.

[61] CABE (2005). Does Money Grow on Trees?.

[62] Smith D. (2010). Valuing Housing and Green Spaces: Understanding Local Amenities, the Built Environment and House Prices in London.

[63] Maantay J.A., Maroko A.R. (2018). Brownfields to greenfields: Environmental justice versus environmental gentrification. Int. J. Environ. Res. Public Health.

[64] Machline E., Pearlmutter D., Schwartz M., Pech P. (2020). Green Neighbourhoods and Eco-Gentrification: A Tale of Two Countries.

[65] Greenspace Information for Greater London (GIGL) (2022). Open Spaces. https://www.gigl.org.uk/our-data-holdings/open-spaces/.

[66] Greater London Authority (2020). Access to Public Open Space by Ward. https://data.london.gov.uk/dataset/access-public-open-space-and-nature-ward.

[67] Greater London Authority (2020). Coronavirus (COVID-19) Cases. https://data.gov.uk/dataset/992f3d28-d917-485f-8c7e-8fd47594a554/coronavirus-covid-19-cases.

[68] Cronk B.C. (2019). How to Use SPSS®: A Step-by-Step Guide to Analysis and Interpretation.

[69] Ntounis N., Saga R.S., Loronõ-Leturiondo M., Hindmarch T., Parker C., The Time to Act Is Now: A Framework for Post-COVID-19 Recovery for Our Towns and Cities (2020). Institute of Place Management (IPM) Blog. http://blog.placemanagement.org/2020/04/02/the-time-to-act-is-now-a-framework-for-post-covid-19-recovery-forour-towns-and-cities/.

[70] Meerow S., Newell J.P. (2019). Urban resilience for whom, what, when, where, and why?. Urban Geogr..

[71] McDonald J. (2014). Fisher’s Exact Test of Independence, BioStatHandbook. http://www.biostathandbook.com/fishers.html.

